# Direct Reprogramming of Mouse Subchondral Bone Osteoblasts into Chondrocyte-like Cells

**DOI:** 10.3390/biomedicines10102582

**Published:** 2022-10-14

**Authors:** Meihan Li, Lingzhi Zhang, Jing Li, Qing Zhu

**Affiliations:** State Key Laboratory of Bioactive Substance and Function of Natural Medicines, Institute of Materia Medica, Chinese Academy of Medical Sciences and Peking Union Medical College, Beijing 100050, China

**Keywords:** cellular reprogramming, transcription factor, osteoblast, chondrocyte, c-Myc, Plagl1, Sox5, Sox9

## Abstract

Treatment of full-thickness articular cartilage defects with exposure of subchondral bone often seen in osteoarthritic conditions has long been a great challenge, especially with a focus on the feasibility of in situ cartilage regeneration through minimally invasive procedures. Osteoblasts that situate in the subchondral bone plate may be considered a potentially vital endogenous source of cells for cartilage resurfacing through direct reprogramming into chondrocytes. Microarray-based gene expression profiles were generated to compare tissue-specific transcripts between subchondral bone and cartilage of mice and to assess age-dependent differences of chondrocytes as well. On osteoblast cell lines established from mouse proximal tibial subchondral bone, sequential screening by co-transduction of transcription factor (TF) genes that distinguish chondrocytes from osteoblasts reveals a shortlist of potential reprogramming factors exhibiting combined effects in inducing chondrogenesis of subchondral bone osteoblasts. A further combinatorial approach unexpectedly identified two 3-TF combinations containing Sox9 and Sox5 that exhibit differences in reprogramming propensity with the third TF c-Myc or Plagl1, which appeared to direct the converted chondrocytes toward either a superficial or a deeper zone phenotype. Thus, our approach demonstrates the possibility of converting osteoblasts into two major chondrocyte subpopulations with two combinations of three genes (Sox9, Sox5, and c-Myc or Plagl1). The findings may have important implications for developing novel in situ regeneration strategies for the reconstruction of full-thickness cartilage defects.

## 1. Introduction

Articular cartilage (AC) is a specialized hyaline cartilage composed of chondrocytes (ACCs) scattered in the spatially organized extracellular matrix (ECM) with histologically distinct zones, an upper superficial zone (SZ) and a subjacent deeper zone (DZ), the latter of which spans the transitional and radial areas. The SZ, covered by synovial fluids, contains flattened chondrocytes (SZCs) with the capability to produce the proteoglycan 4 (Prg4)-encoded lubricin glycoprotein secreted onto the articular surface, providing boundary lubrication [1]. In mice, SZCs are found to be self-renewable as a chondrocyte unipotent progenitor with potential to form cartilage [2]. The deeper zone chondrocytes (DZCs), spherical in shape, are de facto trapped in lacunae and interspersed by an extracellular matrix rich in randomly organized type II collagen with a high content of aggrecan and hyaluronic acid [3]. Within DZ, the terminally differentiated DZCs show no differences in their gene expression pattern and have a minimum of proliferative and migratory capacity [4] and limited effect on extracellular matrix turnover [5].

Damage to the cartilage is often induced directly by mechanical injury or as a consequence of extracellular matrix degradation processes seen most in degenerative or inflammatory disease, leading to various degrees of lesions that could induce depth-dependent responses during cartilage repair. Mechanical attrition that may cause matrix disruption at the cartilage surfaces is likely replaced by the high proliferative and migratory SZCs and newly synthesized matrix [6,7]. When partial or full-thickness chondral defects occur, cartilage can no longer repair itself due to the sparsity and limited reparative capacity of DZCs. If left untreated, the chondral lesions usually enlarge over time, unless injuries extend to the underlying subchondral bone severely enough to elicit a bone marrow-mediated healing process. Homing of endogenous bone marrow-derived stem cells onto the subchondral bone surface enables filling the wound with a mechanically inferior fibrocartilaginous scar [8]. On the basis of this principle, microfracture surgery aimed to provide an enriched microenvironment by artificially creating tiny fractures or holes in the subchondral bone to facilitate cartilage repair by recruited bone marrow cells. Although most frequently applied to any kind of chondral lesion, the inferior biomechanical properties of fibrocartilage infill and inability to sustain long-term function are also concerns [9,10,11].

Autologous chondrocyte implantation, osteochondral autograft transplantation, and microfracture are shown to help prevent or delay the need for joint replacement surgery in some studies [12]. It is suggested that autologous chondrocyte implantation may promote hyaline-like cartilage formation, while osteochondral autograft transplantation (mosaicplasty) could perhaps enable immediate reconstruction with mature AC, producing great clinical outcomes compared with microfracture [9,11]. However, insufficient ACC supply and potential risks of acquiring dedifferentiation phenotype as well as developing considerable donor site morbidity limit the full use of autologous healthy AC and AC-derived chondrocytes [13,14,15]. Cellular reprogramming from one type of cell to another by ectopic expression of biological factors provides the possibility of using stem and specialized cells as additional sources for therapeutic purposes. Conversion into ACC-like cells has been investigated in a wide range of skeletal and nonskeletal cells, such as tenocytes [16], epithelial cells [17], dermal fibroblasts [17,18], stem cell-like embryonic fibroblasts [19], placental cells [20], and embryonic or mesenchymal stem cells [17]. These approaches are usually costly and require lengthy in vitro manipulation of cells with extensive documentation and stringent regulations.

In fact, in the nearest vicinity of the full-thickness chondral lesion, the only functional cells other than chondrocytes are osteoblasts, abundant on the subchondral bone surface during the process of bone remodeling, which occurs continuously. These subchondral bone osteoblasts (SBOs) develop from a common mesenchymal osteochondroprogenitor, as do chondrocytes [21], and chondrocyte-to-osteoblast transdifferentiation fate has been identified during embryonic, post-natal endochondral bone formation as well as during fracture repair in adults [22,23]. This confers a cellular and molecular basis for the concept that SBOs may represent an appropriate new endogenous source of cells for in situ chondrogenic repairs in a more direct and efficient way. Moreover, SBOs are also abundant and easily accessible from differentiated progenitors in marrow bone, promoting new bone formation adjacent to the lesion sites [24]. This suggests more active bone-forming osteoblasts as seed cells that could potentially be utilized. We thus hypothesized that conversion of SBOs directly into articular chondrocyte-like cells could possibly happen by using a certain group of ACC-specific transcription factors. To investigate this, we performed a sequential screening of transcription factors based on differential gene expression profiles from microarray and PCR data. Two sets of three transcription factors (Sox9, Sox5, and c-Myc or Plagl1) were identified to be able to reprogram SBOs to chondrocyte subpopulations, which apparently resemble SZCs and DZCs, respectively. This is a proof of concept that may provide the possibility of developing novel therapeutic strategies for SBO-based regeneration of neocartilage to restore the full-thickness cartilage defect in situ.

## 2. Materials and Methods

### 2.1. Animals and Reagents

C57BL/6 female mice at 5 days (d), 10 weeks (w), and 24 w of age were obtained from Institute of Laboratory Animal Sciences (CAMS&PUMC, Beijing, China). All experimental protocols were approved by the Institute of Materia Medica Animal Authorities (No. 00003787, 27 May 2019).

Antibodies to the following antigens were used: aggrecan (Millipore, AB1031, Burlington, MA, USA), CD44 (BioLegend, 103028, San Diego, CA, USA), Chondroitin Sulfate (Abcam, ab11570, Cambridge, UK), collagen I (Abcam, ab34710, Cambridge, UK), collagen II (Santa Cruz, sc-52658, Dallas, TX, USA), collagen IX (Santa Cruz, sc-376969, Dallas, TX, USA), collagen XI (Lifespan, LS-C-352032, Seattle, WA, USA), hyaluronan binding protein (Millipore, 385911, Burlington, MA, USA), osteocalcin (Santa Cruz, sc-376835, Dallas, TX, USA), syndecan-1 (BioLegend, 142511, San Diego, CA, USA), and donkey anti-rabbit (Jackson, 34213ES60, West Grove, PA, USA) and goat anti-mouse IgG2b (Thermo, A-21144, Waltham, MA, USA).

### 2.2. Cells

Primary subchondral bone (SB) osteoblasts (SBOs) were obtained by outgrowth from explants of proximal tibia SB of 10-w-old mice as previously described [25], with minor modifications. Briefly, the overlying articular cartilage (AC) was removed from the tibial plateau and trabecular bone tissue, then dissected away from the SB plate using a stereomicroscope. The SB samples were minced into small pieces and sequentially digested in the presence of 1 mg/mL type I collagenase (Sigma Aldrich, St. Louis, MO, USA) in α-MEM at 37 °C for 20 min followed by two changes of collagenase for an additional 20 min and 240 min incubation, respectively. After multiple washes with DPBS, the digested bone pieces were placed in flasks and cultured in α-MEM medium containing 20% fetal bovine serum (FBS, Hyclone, Logan, UT, USA). The medium was replaced every day until cells were observed. Cells were then maintained in the medium containing 10% FBS and passaged once at a ratio of 2 × 10^4^ cells/cm^2^ after reaching confluence.

To establish an SBO cell line (SBOL), passage-1 of primary SBOs was seeded in a 24-well plate and co-transfected with piggyBac vector MPH86, which expresses SV40 T Ag flanked with FLP, and the piggyBac transposase expression vector, Super PiggyBac [26]. Stable SBOLs were selected by 10 μg/mL hygromycin B (Roche, Switzerland) in culture for 3 w. Subclones were obtained by limiting dilution. Cells were plated at a density of one cell per well in a 96-well plate and grown for 2–3 w. Colonies were then harvested and expanded. Cells were identified with osteoblast-related markers using PCR analysis, immunohistochemistry, and immunofluorescence.

Isolation of primary chondrocytes from 5 d, 10 w, and 24 w old mice was described elsewhere [27,28]. In brief, after manual macrodissection of the femoral condyles and tibial plateau, adherent soft tissues were removed from the cartilage by digestion with 3 mg/mL collagenase D (Roche, Switzerland) for 45 min × 2 times at 37 °C. The cartilage was then enzymatically digested with 0.5 mg/mL collagenase D solution overnight at 37 °C to release chondrocytes. The isolated cells were collected by centrifugation and resuspended in a fresh medium and seeded onto plates or dishes containing DMEM with 10% FBS. Culture cells were identified with chondrocyte-related markers using immunohistochemistry and immunofluorescence. To isolate hyaline cartilage from neonatal mice, femoral heads, femoral condyles and tibial plateau were removed using a scalpel [27]. The connective tissues were removed by collagenase D digestion. To isolate hyaline cartilage from young and adult mice, the entire cartilage was removed and cut free from any brownish tissue (calcified cartilage). To isolate the subchondral bone, the full-thickness cartilage including the calcified zone was removed and cut free from any brownish tissue to expose the subchondral bone.

### 2.3. Microarray Analysis

Tissues from 5 mice and cells from 5–10 mice were pooled into RNase-free microcentrifuge tubes. The remaining tissues were stained with Safranin-O for quality control. AC and SB were pulverized using a liquid nitrogen-cooled tissue grinder prior to RNA isolation. Total RNA was extracted from each sample using TRIzol (Invitrogen, Waltham, MA, USA) and purified. RNA amplification, labeling, and hybridization were conducted by CapitalBio Technology (Beijing, China) using the GeneChip Mouse Genome 430 2.0 Array (Affymetrix, Santa Clara, CA, USA). Fold change was calculated by normalizing signal intensities to the corresponding AC or 5-d-old ACC controls. A more than 2-fold change with *p* < 0.05 was considered as differentially regulated genes.

### 2.4. Lentivirus Vectors and Transduction

Candidate cDNA was cloned into lentiviral transducing vectors using primers shown in Table S1. One day after seeding 293T cells at 3 × 10^6^ cells per 10-cm dish, we transfected the cells with PsPAX2, pMD2.G plasmid and pSIN4-based lentiviral vectors (Addgene, Watertown, MA, USA) using Lipofectamine 3000 (Invitrogen, Waltham, MA, USA). The virus-containing supernatants were collected at 48 h and 72 h post-transfection and filtered through 0.45-µm cellulose acetate filters (Millipore, Burlington, MA, USA). Lentiviral particles were concentrated by mixing with Lenti-Concentin Virus Precipitation Solution (ExCell Biology, China). Viral concentration was determined by HIV-1 p24 Antigen Capture Assay (Abcam, Cambridge, UK). Viruses were stored at −80 °C until use.

SBOLs were co-transduced with lentiviruses of transcription factors twice with 8 µg/mL polybrene (Sigma Aldrich, St. Louis, MO, USA) in FBS and antibiotic-free medium. After 12 h incubation, the virus-containing medium was replaced with a primary chondrocyte culture medium. At d14 post transduction, transduced cells were harvested and characterized for chondrogenic differentiation.

### 2.5. Laser Capture Microdissection (LCM) and Quantitative PCR

Cells transduced with lentivirus encoding transcription factors were cultured in PEN Membrane dish (Leica, 11505172, Wetzlar, Germany), and fixed with 70% ethanol for 15–30 s. After washing with DEPC-treated DPBS for 15 s, cells were permeabilized with 0.5% Triton X-100 for 3 min. Cells were incubated with primary antibodies (collagen II and aggrecan) overnight, followed by fluorescently labeled secondary antibodies, and counterstained with DAPI. Double positive cells were laser capture microdissected with a Leica LMD7000 system (Leica, Wetzlar, Germany). Captured cells in the 0.5 mL PCR cap were mixed in the tube containing 65 μL lysis buffer and centrifuged before being stored at −80 °C. Total RNA was extracted from the microdissected cells by the RNeasy Micro Kit (Qiagen, 74004, Hilden, Germany) for one-step RT-qPCR using a Single Cell Sequence Specific Amplification Kit (Vazyme, P621-01, Nanjing, China) according to the manufacturer’s protocol.

Total RNA of ACCs or established SBOLs was extracted using TRIzol and reverse transcribed to cDNA using SuperScript II reverse transcriptase following the manufacturer’s protocol (Toyobo, Osaka, Japan). RT-qPCR was performed on StepOnePlus Real-Time PCR System with SYBR Green PCR Master Mix (TransGen Biotech, China). All signals were normalized to that for *Actb*, and gene expression was compared using the 2^−∆∆Ct^ method. Primer sequences used for RT-qPCR are listed in Table S2.

### 2.6. Immunohistochemistry and Immunofluorescence

AC or SB specimens were fixed in 4% paraformaldehyde and then routinely processed for paraffin embedding. Tissues were cut into sections from the paraffin blocks and stained with Safranin-O/Fast Green for glycosaminoglycans.

Cell samples were fixed with 4% paraformaldehyde and permeabilized with 0.3% Triton X-100 for 10 min. After 30 min of 1% BSA blocking, cells were incubated with primary antibodies at 4 °C overnight, followed by incubation with appropriate fluorescently labeled secondary antibodies for 1 h at RT. Cell nuclei were counterstained with DAPI (Macgene, CD051, Beijing, China). Images were taken by Operetta High Content Analysis (PerkinElmer, Waltham, MA, USA).

### 2.7. Osteogenesis Assay (Alkaline Phosphatase and Alizarin Red S)

Cells were fixed with 4% paraformaldehyde for 15 min and then stained using the alkaline phosphatase kit in accordance with the manufacturer’s instructions (Beyotime Biotechnology, Shanghai, China).

After 12 d of osteogenic differentiation, cells were fixed with 4% paraformaldehyde for 15 min at RT and then washed with DPBS. Cells were then stained with 1% Alizarin Red S (Solarbio, Beijing, China) for 30 min at 37 °C and washed three times with distilled water.

### 2.8. Flow Cytometric Analysis

Cells were resuspended in 100 µL of DPBS containing 0.1% BSA and incubated with anti-CD44 or anti-syndecan-1 antibodies in the dark for 1 h at 4 °C. A secondary antibody was used for syndecan-1 staining. Sample data were acquired on a FACSVerse (BD, Sunnyvale, CA, USA) and analyzed with FlowJo software (TreeStar Inc., Ashland, OR, USA).

### 2.9. Statistics

Comparisons between groups were analyzed by Student’s t-test. Comparisons among means of more than two groups were determined by one-way ANOVA post hoc with Bonferroni correction. A *p* value less than 0.05 was considered statistically significant.

## 3. Results

Articular chondrocytes possess a gene expression signature distinct from subchondral bone osteoblasts.

**Figure 1:**
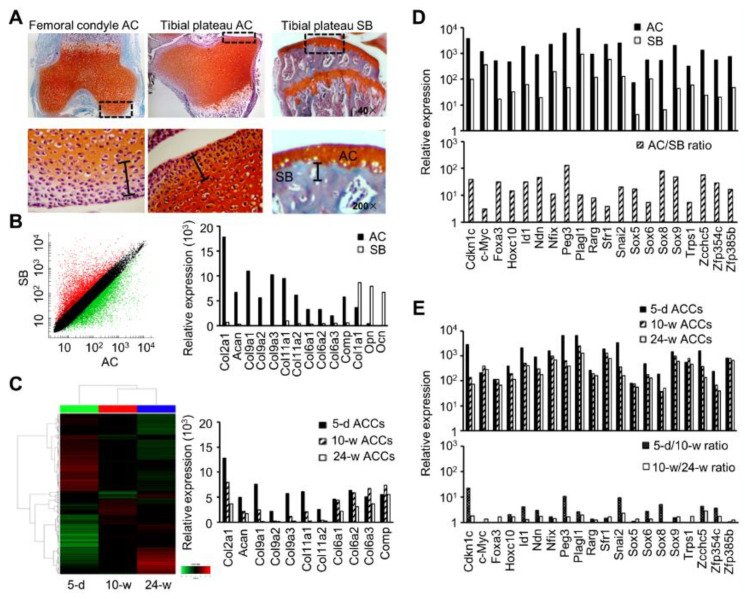
Transcriptome analysis reveals tissue- and age-specific gene expression. Articular cartilage (AC) and subchondral bone (SB) tissues were obtained from knee cartilage and SB plate, respectively, and chondrocytes were isolated from knee cartilage using collagenase D. Total RNA was extracted from tissue and cell samples prior to microarray analysis. (**A**) Dissection of knee AC and SB stained with Safranin-O/Fast Green. Magnification: 40× (upper row) and 200× (lower row). (**B**) Scatter plot of differentially expressed genes (left) and expression of cell type-specific genes (right). Samples of AC and SB are compared. (**C**) Heatmap showing hierarchical clustering of samples with at least 2-fold differences in gene expression (left) and gene expression of transcription factors of interest (right). Samples from AC chondrocytes (ACCs) of 5 d, 10 w and 24 w are compared. Red and green intensities denote high and low levels of gene expression, respectively. (**D**) Transcription factors highly expressed in AC compared to SB. (**E**) Highly expressed transcription factors in ACCs of 5 d, 10 w and 24 w old mice. A list of abbreviations is in Table S3.

In order to determine the transcription factors that may have the potential to convert subchondral bone (SB) osteoblasts (SBOs) to the fate of articular cartilage (AC) chondrocytes (ACCs), we first performed microarray analyses comparing gene expression levels between AC and SB as well as ACCs from different ages of mice. Using manual macrodissection techniques, AC covering the knee joint and SB underneath were harvested from mice, followed by frozen tissue grinding or cell isolation (Figure 1A). Between the two types of tissues, there were 6097 differentially expressed genes with ≥ 2-fold difference, among which 3631 were higher and 2466 lower in AC than in SB. The collagen type II protein-encoding gene *Col2a1* was most abundantly expressed in AC over other extracellular matrix-associated genes, including *Acan*, *Col9a1–3*, *Col11a1 and a2*, *Col6a1–3*, and *Comp*, all highly represented compared to those in SB. In contrast, *Col1a1* and the noncollagenous matrix genes *Opn* and *Ocn* were more expressed in SB than in AC (Figure 1B). Thus, AC and SB had strikingly distinct patterns of gene expression pertaining to extracellular matrix.

It has been shown that ACCs have a dramatic age-related decline in chondrogenic potential with significant phenotypic changes [29]. Age-associated gene expression patterns were determined from a three-sample comparison of ACCs between neonatal, young, and old mice. The highly expressed *Col2a1* and *Acan* in neonatal ACCs were decreased with age and remained significant in old cells (Figure 1C), underlying the specific traits of ACCs. *Col9a1–3* and *Col11a1* and *a2* at high levels in neonatal ACCs declined dramatically in young and became undetectable in elderly cells, suggestive of an association of these genes with early stages of chondrogenesis. With respect to *Col6a1–3* and *Comp*, no significant age-related differences in expression were observed (Figure 1C). As these genes are shown to play an essential role in the ossification process [30,31], they were not included within the scope of the evaluation. The results might underscore a possible link between age-associated expression pattern and direct reprogramming into chondrocytes. This implied that the corresponding matrix proteins type II and aggrecan might be mainly used to determine the SBO-to-ACC conversion efficiency.

Our data demonstrated that among 235 transcription factor (TF) genes, 20 of them had a higher level in AC than in SB (Figure 1D), including those with decreased expression with age (Figure 1E). We then investigated TFs differentially expressed between AC and SB as well as changes with age in chondrocytes. Specifically, *Ndn*, *Zcchc5*, and *Zfp354c* had age-related expression profiles with a steady and strong decline, and *Cdkn1c*, *Id1*, *Peg3*, *Snail2*, and *Sox8* dropped sharply in young but with no further reduction in elderly cells. Moreover, *Hoxc10*, *Nfix*, *Plagl1*, *Rarg*, *Sfr1*, *Sox6*, and *Sox9* experienced a slight decrease with age, and minimal changes occurred to *c-Myc*, *Foxa3*, *Sox5*, *Trps1*, and *Zfp385b* at all ages (Figure 1E). We believed that an understanding of these TF genes, either strongly or weakly associated with age, might help gain mechanistic insights into the chondrogenic reprogramming process.

### 3.1. Transformed SBOs Preserve Their Original Phenotypes

**Figure 2:**
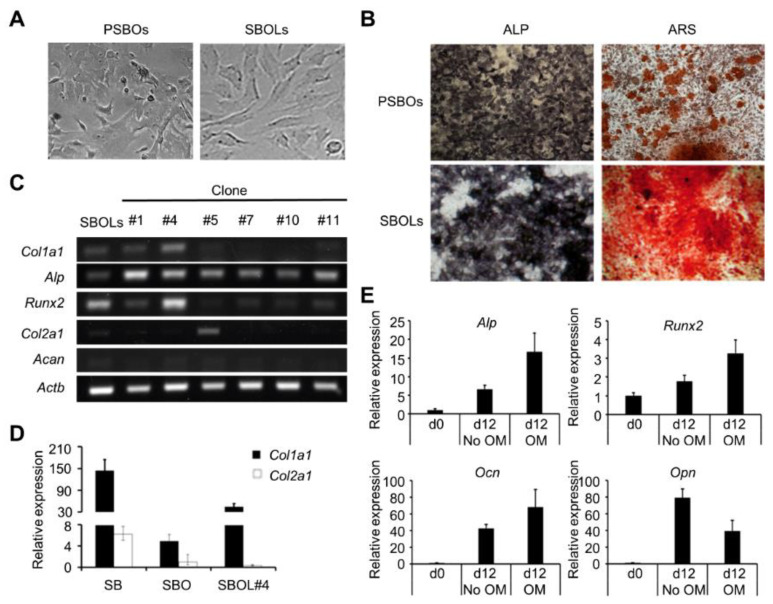
Established SBOLs maintain the primary osteoblastic phenotype. Stable subchondral bone osteoblasts (SBOLs) were selected in the presence of 10 μg/mL hygromycin B following immortalization of primary SBO (PSBO) cells by co-transfection with SV40 T Ag and piggyBac transposase expression vector. For the mineralization assay, cells were grown for 12 d in the presence (+) or absence (−) of the osteogenic medium (OM). (**A**) Light microscopy of PSBOs and SBOLs. Magnification: 100×. (**B**) Alkaline phosphatase (ALP) and Alizarin Red S (ARS) staining of PSBOs and SBOLs cultured for 7 and 12 d, respectively. Magnification: 40×. (**C**) PCR analysis of gene expression in SBOL subclones. (**D**) Levels of *Col1a1* and *Col2a1* expression in SB, PSBOs and SBOL clone #4 by RT-qPCR. (**E**) Measurement of *Alp*, *Runx2*, *Ocn*, and *Opn* expression in SBOL clone #4 cultured under osteogenic conditions. Data shown in bar graphs represent mean ± SD (*n* = 3).

Due to the scanty supply of primary SBOs, we established a subchondral bone osteoblast cell line (SBOL) for subsequent screening assays. Mouse primary SBOs (PSBOs) were immortalized by introducing SV40, resulting in elongated or spindle-shaped morphology like PSBOs (Figure 2A and Figure S1A) and producing high levels of alkaline phosphatase (Figure 2B). We then characterized six subclonal SBOLs, among which clones 1, 4, and 11 had levels of alkaline phosphatase and mineralization similar to those of the PSBOs (Figure S1B). However, clone 4 showed significant mRNA levels of all three tested osteoblast-related genes *Col1a1, Alp*, and *Runx2* but undetectable levels of chondrocyte-related genes *Col2a1* and *Acan* (Figure 2C). The *Col1a1* expression was essentially high although still lower than freshly isolated SB tissue (Figure 2D). Immunofluorescence staining confirmed that clone 4 expressed bone-specific matrix proteins COL1A1 as well as OCN (Figure S1C). Further characterization of clone 4 showed markedly upregulated *Alp, Runx2*, and *Ocn* with a decreased level of *Opn* (Figure 2E). This was in agreement with previous findings that *Opn* progressively decreased after an initial 7 d of elevated expression relative to *Ocn* [32]. Since the SBOL clone 4 showed phenotypes very similar to those of PSBOs, it was then employed as a surrogate for subsequent TF screening.

### 3.2. Combinatorial Function of Ectopic Transcription Factors Is Required for Direct Reprogramming of SBOLs into ACCs

**Figure 3:**
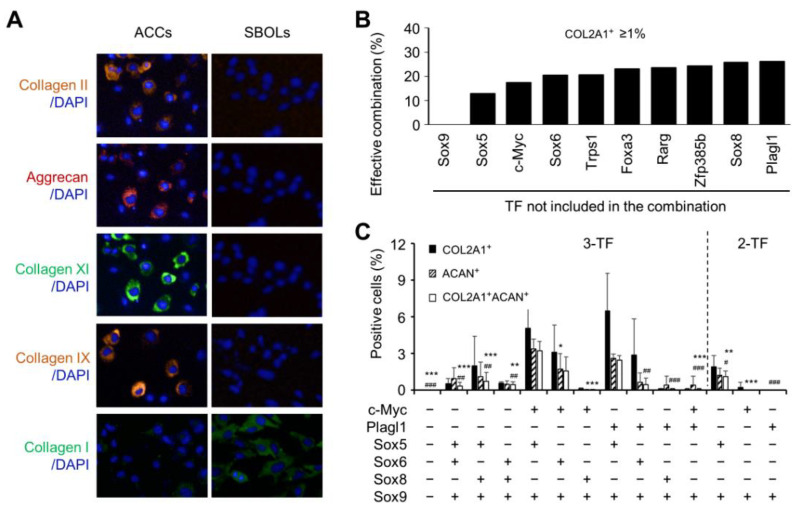
Ectopic expression of Sox9 and Sox5 together with c-Myc or Plagl1 directly converts SBOs into ACC-like cells. SBOLs were transduced with transcription-expressing lentiviruses at an MOI of 100 twice with 8 µg/mL polybrene. After 14 d of culture, cells were fixed for immunofluorescence analyses. (**A**) Immunofluorescence staining of collagen and aggrecan in primary ACCs and SBOLs. Magnification: 200×. (**B**) Transcription factors essential in the reprogramming of SBOs into ACC-like cells, which was determined based on the efficiency in inducing COL2A1 expression by all of the combinations that did not include the transcriptional factor indicated. Each one was tested in at least 20 different combinations containing 4 or 6 transcription factors. (**C**) Induction of COL2A1^+^, ACAN^+^, and COL2A1^+^ACAN^+^ cells by Sox9-containing 2–3-TF combinations. Data shown in the bar graph represent mean ± SD (*n* = 3–4). *, **, and *** indicate statistically significant difference from Sox9 + Sox5 + c-Myc. #, ##, and ### indicate statistically significant difference from Sox9 + Sox5 + Plagl1.

Hyaluronan (HA), chondroitin sulfate (CS), syndecan-1, and CD44 were highly expressed in chondrocytes, but also abundant in osteoblasts (Figure S2A,B). These surface molecules were not chondrocyte-specific and hence not sufficient to distinguish chondrocytes from osteoblasts. Indeed, types II, IX and XI collagen and aggrecan were preferentially produced in ACCs, whereas type I collagen was primarily expressed in SBOLs (Figure 3A). The data indicated that chondrocyte-specific extracellular matrix collagens and proteoglycan could be used as phenotypic markers for functional screening of the transcription factors that may have prochondrogenic potential to generate transcription factor-converted ACCs (cACCs). We then tested whether expression of a single transcription factor was sufficient to confer chondrogenic potential onto SBOLs. Figure S3A ruled out the possibility of transfecting each of the 20 candidate transcription factors to induce SBOLs to express either type II collagen or aggrecan.

We then proceeded to determine the minimum number of transcription factors needed to induce ACC-specific genes by screening 6 transcription factor (6-TF) combinations. The results indicated that 10 transcription factors, which were Sox9, Zfp385b, c-Myc, Foxa3, Plagl1, Sox6, Sox8, Sox5, Trps1, and Rarg, could imbue chondrogenic potential onto SBOLs in more than 10% of 6-TF combinations from all 20 candidate transcription factors (Figure S3B). The 10 transcription factors were next used in experiments using 4–6-TF combinations. The results suggested that Sox9 was indispensable to the conversion, as no type II collagen could be detected in the absence of Sox9 (Figure 3B). This allowed us to further narrow down transcription factors to Sox9-containing 3-TF combinations and assess both type II collagen and aggrecan proteins in treated cells. We found that Sox9 + Sox5-containing 3-TF combinations with c-Myc or Plagl1 induced substantially higher COL2A1⁺ACAN⁺ double-positive cells than Sox9 + Sox6-containing combinations; and the Sox9 + Sox8-containing combination with c-Myc or Plagl1 barely induced double-positive cells (Figure 3C). Further, Sox9 + c-Myc + Plagl1 treatment was not effective at stimulating double-positive cells, suggesting that c-Myc and Plagl1 did not cooperatively enhance the reprogramming efficiency (Figure 3C). Moreover, Sox9 in combination with c-Myc or Plagl1 (2-TF without Sox5) did not show significant effects, either. Of note, Sox9 and Sox5 in combination with c-Myc or Plagl1 tended to induce more COL2A1^+^ than ACAN^+^ cells (Figure 3C), in agreement with previous findings showing more type II collagen than aggrecan produced in chondrocytes [33]. Furthermore, both 3TF combinations could induce types IX and XI collagen expression (Figure S3C). We confirmed that the Sox9 + Sox5 combination was inefficient at inducing expression of type II collagen and aggrecan as well as types IX and XI collagen (Figure S3D). Taken together, Sox9 and Sox5 in combination with Plagl1 or c-Myc each represent the minimum number of transcription factors with the potential to confer chondrogenic potential onto osteoblasts.

### 3.3. Stable Expression of Plagl1 Improves the Efficiency of Reprogramming to cACCs

**Figure 4:**
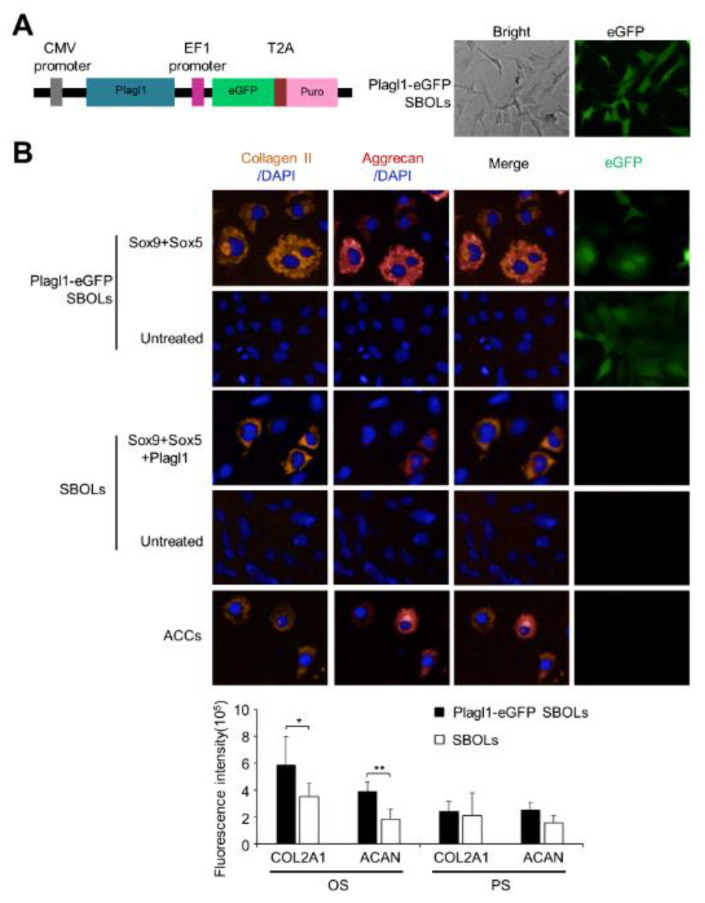
SBOs can be converted into two cell populations phenotypically resembling superficial and deeper zone ACCs, respectively. SBOLs were transduced with pCDH-Plagl1-eGFP lentiviruses and selected with 1.5 μg/mL puromycin for 2 w to generate Plagl1-eGFP SBOLs, resulting in more than 80% of eGFP-positive cells. Plagl1-eGFP SBOLs were then transduced with SOX9 and Sox5 lentiviruses at an MOI of 100. After 14 d culture, transfected cells were fixed for immunofluorescence analyses. (**A**) Establishment of Plagl1-eGFP SBOLs. *Left panel*: Schematic representation of the transgene. *Right panel*: Fluorescence microscopy of Plagl1-eGFP SBOLs. Magnification: 100×. (**B**) *Upper panel*: Immunofluorescence microscopy of COL2A1^+^, ACAN^+^ and COL2A1^+^ACAN^+^ Plagl1-eGFP SBOLs after transduction with transcription factors indicated. Magnification: 200×. *Lower panel*: Fluorescence intensity of COL2A1 and ACAN expression in oval shaped (OS) and flat polygonal shaped (PS) cells. * *p* < 0.05, ** *p* < 0.01.

As Plagl1 has been shown to be involved in chondrogenic differentiation [34], we established SBOLs that stably expressed eGFP linked to Plagl1 (Plagl1-eGFP SBOLs) and sought to determine its effectiveness in reprogramming SBOs (Figure 4A). Plagl1-eGFP SBOLs treated with Sox9 and Sox5 showed a slight increase in the fluorescence intensity of COL2A1 compared to SBOLs treated with Sox9 + Sox5 + Plagl1 in oval-shaped cells; however, the fluorescence intensity of ACAN in Sox9- and Sox5-treated Plagl1-eGFP SBOLs was markedly increased by 2.1-fold (Figure 4B). Thus, stable expression of Plagl1 significantly enhanced the synthesis of type II collagen and aggrecan in oval-shaped cACCs compared with the flat polygonal-shaped cells, confirming the important role of Plagl1 in conversion of SBOs into mature cACCs.

**Figure 5:**
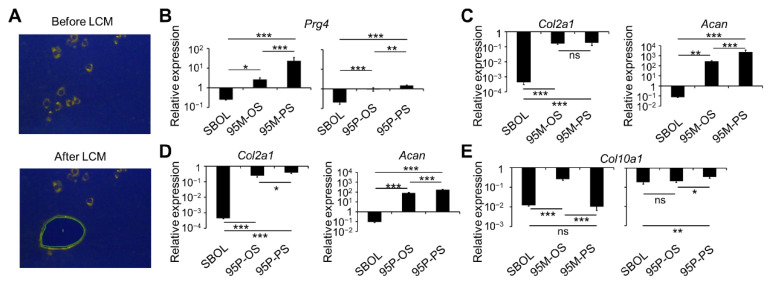
SBO-to-ACC differential conversion can be induced by two sets of transcription factors. SBOs were transfected with Sox9 and Sox5 together with c-Myc (95M) or Plagl1 (95P) and incubated for 14 d prior to the assessment of *Col2a1* and *Acan* expression by RT-qPCR in laser capture microdissected (LCM) cells based on their morphology (OS or PS) and chondrogenic phenotype (positive for both COL2A1 and ACAN examined under fluorescence microscope). (**A**) Immunofluorescence stained samples (photo enhanced) before (*upper*) and after (*lower*) LCM. Magnification: 100×. (**B**) Expression of the superficial zone (SZ)-specific gene *Prg4* in OS and PS cells treated with 95M and 95P. (**C**,**D**) Expression of *Col2a1* and *Acan* in OS and PS cells treated with 95M (**C**) or 95P (**D**). (**E**) Assessment of the hypertrophic chondrocyte-specific marker gene *Col10a1*. Data shown in the bar graph represent mean ± SD (*n* = 3). Individual RNA expression levels were normalized to respective *Actb* expression levels and relative to natural ACCs. SBOLs served as negative control. * *p* < 0.05, ** *p* < 0.01, *** *p* < 0.001.

Finally, we wanted to figure out how two combinations of transcription factors were needed in the conversion of SBOs into ACCs. It is known that the non-calcified ACCs are organized largely into the superficial and deeper (including middle and deep) zones (SZ and DZ, respectively) and there are phenotypic differences in cellular function between the superficial and deeper zone chondrocytes (SZCs and DZCs, respectively). Particularly, SZCs express Prg4, a SZ-specific proteoglycan that will no longer be produced when they expand into DZCs with age [35,36]. We thus assessed the expression of Prg4 for the cACCs induced by Sox9 + Sox5 + c-Myc (95M) and Sox9 + Sox5 + Plagl1 (95P), respectively. Cells transfected with 95M or 95P to co-express COL2A1 and ACAN were isolated by LCM from the plates by virtue of their morphology, i.e., either flat polygon or oval in shape, for RT-qPCR measurement (Figure 5A). 95M-induced flat polygonal-shaped (95M-PS) cACCs had higher expression of *Prg4* than oval-shaped (95M-OS) cACCs (Figure 5B). However, neither 95P-OS nor 95P-PS cACCs showed appreciable levels of Prg4 (Figure 5B). Upregulated *Col2a1* and *Acan* confirmed chondrogenic properties of both types of cACCs with either morphology (Figure 5C,D). Hypertrophic chondrocytes were not induced, as there was no increased expression of *Col10a1* in 95M and 95P cACCs compared to natural ACCs (Figure 5E). We further confirmed that virtually all 95M- and 95P-reprogrammed cells were successfully converted, as indicated by the loss of osteoblast identity (Figure S4). Thus, 95M-PS might resemble SZCs, while 95M-OS could conceivably be intermediate between SZCs and DZCs because of the lower expression of Prg4 and the oval shape. Not only 95P-OS but also 95P-PS most possibly represented DZCs since polygonal cells are also present in DZ. These results indicated that two zonal populations of ACCs were likely to be reprogrammed from SBOs by Sox9 and Sox5 in combination with c-Myc or Plagl1.

## 4. Discussion

In the present study, we demonstrate the ability to convert SBOs into two zone-like cACCs, each induced by transduction of a 3-TF combination of Sox9 and Sox5 with an additional factor, either c-Myc or Plagl1. Sox9 as the master regulator for chondrocyte development was shown in this study to be essential in the SBO-to-ACC conversion, and in the presence of Sox9 and Sox5, c-Myc drives conversion of SBOs toward Prg4⁺ SZ-like cACCs, while Plagl1 favors the reprogramming into mature DZ-like cACCs, which is negative for Prg4.

Sox9 is known to bind to and activate chondrocyte-specific enhancer elements in Col2a1 [37], Col9a1 [38], Col11a2 [39], and aggrecan [40], and is required for chondrogenic mesenchymal condensation in the development of bud limbs [41]. Another important role of Sox9 is to inhibit chondrocyte hypertrophy [42,43]. However, Sox9 alone was not enough to convert SBOs into functional ACCs. We showed that Sox5 and, to a lesser extent, Sox6 played an important role in chondrogenic conversion. It has been shown that their effects on chondrogenic mesenchymal condensation in bud limb development largely depend on the presence of Sox9 [41]. They form homo- and hetero-dimers and cooperatively bind with Sox9, known as the Sox trio, to enhance the expression of *Col2a1* [44] and *Acan* [45]. Although double knockout of Sox5 and Sox6 leads to severe chondrodysplasia [46], lacking either of them has modest skeletal defects, suggestive of genetic redundancy. The two combinations had no need for exogenous Sox6 probably because of its higher endogenous level in SBOs in comparison to Sox5, which might be too low to significantly impact the conversion.

In the fibroblast-to-chondrocyte conversion, c-Myc alone or combined with Klf4 and SOX9 has been shown to be an effective reprogramming factor [18,19,20,47]. It is notable that c-Myc is considered to be a cell cycle driver, and as a putative target of Sox9, its level in prechondrocytes derived from skeletal progenitors is increased [48]. Depending on the severity of osteoarthritis, superficial and transitional zone chondrocytes are induced to upregulate c-Myc expression presumably to regenerate damaged cartilage surfaces. The advantage of overexpression of c-Myc is that chondrocytes are maintained in a proliferative state and their maturation into hypertrophic chondrocytes can be impeded [49].

Identification of the zinc finger transcription factor Plagl1 is fundamental for the understanding of reprogramming of SBOs into mature ACCs. This gene is predominantly localized in developing cartilage and acts as a potential regulator of chondrogenic differentiation. Plagl1 target genes are involved in extracellular matrix receptor binding, collagen binding, cell adhesion, and controlling extracellular matrix secretion and deposition [50]. The distribution of Plagl1 is in close association with that of the Sox trio, Col2a1, and aggrecan during mesenchymal condensation and chondrocyte maturation [34,51]. Its expression persists throughout postnatal stages and continues into adulthood [52]. This is in agreement with our microarray data showing expression of Plagl1 as well as Sox9 in a slow, steady decline with age. Further, we found that overexpression of Plagl1 enhanced Acan expression markedly in oval-shaped cells, which matches the property of DZCs. As Plagl1 functions to suppress cell growth by inducing cell cycle arrest [53], it might help to promote chondrocyte terminal maturation that naturally occurs in the DZ and possibly prevent hypertrophic differentiation as well.

In OA, Sox9 is substantially decreased in expression by inflammatory mediators [54], and transduction of Sox9 is shown to prevent IL-1β-induced chondrocyte apoptosis effectively through inhibition of Smad3 degradation [55]. Increased Sox9 can also inhibit TNF-α and subsequently the NF-κB signaling pathway [56]. As some studies also found weakened expression of Sox5 in OA [57,58], elevation of Sox5 could potentially counteract the inhibitory effects of proinflammatory mediators on the gene. Plagl1 is shown to be capable of upregulating the IL-1β-suppressor IL-11, which is also lowered in OA, suggesting the potential value of Plagl1 as a therapeutic agent for OA cartilage defects [59]. As lubricin exhibits anti-inflammatory effects [60,61], increased expression of Prg4 through transduction of c-Myc may help foster an anti-inflammatory microenvironment for cartilage regeneration. Thus, the reprogramming factors identified in this study might also be implicated in treating OA-associated inflammation.

In the case that the subchondral bone is not fully exposed due to the presence of some calcified cartilage in the lesion, which reportedly inhibits cartilage healing [62], removal of calcified cartilage may be necessary before in situ transduction. However, recent studies demonstrate that preserving calcified cartilage does not have significant inhibitory effects [63]. As direct transdifferentiation of chondrocytes into osteoblasts naturally occurs [64], a question is whether there exist osteoblasts derived from calcified cartilage cells in the deepest calcified zone in response to cartilage injury and, if they do, whether such transdifferentiated osteoblasts can be targeted for direct conversion. However, osteoblasts may not be the only source cells attractive for direct reprogramming into chondrocytes. Osteocytes that are abundant in the subchondral bone may also be a feasible target for the conversion into superficial and deeper zone chondrocytes, which warrants further study.

In conclusion, we have developed a novel strategy with the potential to convert osteoblasts into SZ- and DZ-like chondrocyte populations by two sets of 3-TF combinations, each containing the same two factors Sox9 and Sox5 and a different third one, either c-Myc or Plagl1. Although much refinement and characterization of such transdifferentiating processes warrant further investigation, the findings reported here highlight the potential of in situ conversion of the endogenous osteoblasts situated in the subchondral bone plate, when exposed to the full-thickness chondral lesions, into zonal chondrocytes to regenerate structurally organized neocartilage.

## Data Availability

The data that support the findings of this study are available from the corresponding author upon reasonable request.

## Supplementary Materials

The following supporting information can be downloaded at: https://www.mdpi.com/article/10.3390/biomedicines10102582/s1, Figure S1: SBOLs retained osteoblastic properties; Figure S2: ACCs and SBOLs shared some indistinguishable features excluded for the characterization of cACCs; Figure S3: Sox9 and Sox5 together with c-Myc or Plagl1 as 3-TF combination was essential for cACCs generation; Figure S4: 95M- and 95P-reprogrammed SBOLs lost their osteoblast identity; Table S1: Sequences of primers used for lentivirus construction; Table S2: Sequences of primers used in PCR reactions; Table S3: Abbreviations.

## References

[1] Saito T. (2022). The superficial zone of articular cartilage. Inflamm. Regen..

[2] Li L., Newton P.T., Bouderlique T., Sejnohova M., Zikmund T., Kozhemyakina E., Xie M., Krivanek J., Kaiser J., Qian H. (2017). Superficial cells are self-renewing chondrocyte progenitors, which form the articular cartilage in juvenile mice. FASEB J..

[3] Carballo C.B., Nakagawa Y., Sekiya I., Rodeo S.A. (2017). Basic Science of Articular Cartilage. Clin. Sport. Med..

[4] Amanatullah D.F., Yamane S., Reddi A.H. (2014). Distinct patterns of gene expression in the superficial, middle and deep zones of bovine articular cartilage. J. Tissue Eng. Regen. Med..

[5] Singh P., Marcu K.B., Goldring M.B., Otero M. (2019). Phenotypic instability of chondrocytes in osteoarthritis: On a path to hypertrophy. Ann. N. Y. Acad. Sci..

[6] Seol D., McCabe D.J., Choe H., Zheng H., Yu Y., Jang K., Walter M.W., Lehman A.D., Ding L., Buckwalter J.A. (2012). Chondrogenic progenitor cells respond to cartilage injury. Arthritis Rheum..

[7] Shiromoto Y., Niki Y., Kikuchi T., Yoshihara Y., Oguma T., Nemoto K., Chiba K., Kanaji A., Matsumoto M., Nakamura M. (2022). Increased migratory activity and cartilage regeneration by superficial-zone chondrocytes in enzymatically treated cartilage explants. BMC Musculoskelet. Disord..

[8] Tesche F., Miosge N. (2005). New aspects of the pathogenesis of osteoarthritis: The role of fibroblast-like chondrocytes in late stages of the disease. Histol. Histopathol..

[9] Riboh J.C., Cvetanovich G.L., Cole B.J., Yanke A.B. (2017). Comparative efficacy of cartilage repair procedures in the knee: A network meta-analysis. Knee Surg. Sport. Traumatol. Arthrosc..

[10] Shimozono Y., Coale M., Yasui Y., O’Halloran A., Deyer T.W., Kennedy J.G. (2018). Subchondral Bone Degradation After Microfracture for Osteochondral Lesions of the Talus: An MRI Analysis. Am. J. Sport. Med..

[11] Solheim E., Hegna J., Strand T., Harlem T., Inderhaug E. (2018). Randomized Study of Long-term (15–17 Years) Outcome After Microfracture Versus Mosaicplasty in Knee Articular Cartilage Defects. Am. J. Sport. Med..

[12] Knutsen G., Drogset J.O., Engebretsen L., Grontvedt T., Ludvigsen T.C., Loken S., Solheim E., Strand T., Johansen O. (2016). A Randomized Multicenter Trial Comparing Autologous Chondrocyte Implantation with Microfracture: Long-Term Follow-up at 14 to 15 Years. J. Bone Jt. Surg..

[13] Malicev E., Kregar-Velikonja N., Barlic A., Alibegovic A., Drobnic M. (2009). Comparison of articular and auricular cartilage as a cell source for the autologous chondrocyte implantation. J. Orthop. Res..

[14] Andrade R., Vasta S., Pereira R., Pereira H., Papalia R., Karahan M., Oliveira J.M., Reis R.L., Espregueira-Mendes J. (2016). Knee donor-site morbidity after mosaicplasty—A systematic review. J. Exp. Orthop..

[15] Li M.H., Xiao R., Li J.B., Zhu Q. (2017). Regenerative approaches for cartilage repair in the treatment of osteoarthritis. Osteoarthr. Cartil..

[16] Takimoto A., Oro M., Hiraki Y., Shukunami C. (2012). Direct conversion of tenocytes into chondrocytes by Sox9. Exp. Cell Res..

[17] Ikeda T., Kamekura S., Mabuchi A., Kou I., Seki S., Takato T., Nakamura K., Kawaguchi H., Ikegawa S., Chung U.I. (2004). The combination of SOX5, SOX6, and SOX9 (the SOX trio) provides signals sufficient for induction of permanent cartilage. Arthritis Rheumatol..

[18] Hiramatsu K., Sasagawa S., Outani H., Nakagawa K., Yoshikawa H., Tsumaki N. (2011). Generation of hyaline cartilaginous tissue from mouse adult dermal fibroblast culture by defined factors. J. Clin. Investig..

[19] Shi J.W., Zhang T.T., Liu W., Yang J., Lin X.L., Jia J.S., Shen H.F., Wang S.C., Li J., Zhao W.T. (2019). Direct conversion of pig fibroblasts to chondrocyte-like cells by c-Myc. Cell Death Discov..

[20] Ishii R., Kami D., Toyoda M., Makino H., Gojo S., Ishii T., Umezawa A. (2012). Placenta to cartilage: Direct conversion of human placenta to chondrocytes with transformation by defined factors. Mol. Biol. Cell.

[21] Zou L., Zou X., Li H., Mygind T., Zeng Y., Lu N., Bunger C. (2006). Molecular mechanism of osteochondroprogenitor fate determination during bone formation. Adv. Exp. Med. Biol..

[22] Yang L., Tsang K.Y., Tang H.C., Chan D., Cheah K.S. (2014). Hypertrophic chondrocytes can become osteoblasts and osteocytes in endochondral bone formation. Proc. Natl. Acad. Sci. USA.

[23] Aghajanian P., Mohan S. (2018). The art of building bone: Emerging role of chondrocyte-to-osteoblast transdifferentiation in endochondral ossification. Bone Res..

[24] Thambyah A., Broom N. (2009). On new bone formation in the pre-osteoarthritic joint. Osteoarthr. Cartil..

[25] Hilal G., Martel-Pelletier J., Pelletier J.P., Ranger P., Lajeunesse D. (1998). Osteoblast-like cells from human subchondral osteoarthritic bone demonstrate an altered phenotype in vitro: Possible role in subchondral bone sclerosis. Arthritis Rheum..

[26] Wang N., Zhang W., Cui J., Zhang H., Chen X., Li R., Wu N., Chen X., Wen S., Zhang J. (2014). The piggyBac transposon-mediated expression of SV40 T antigen efficiently immortalizes mouse embryonic fibroblasts (MEFs). PLoS ONE.

[27] Gosset M., Berenbaum F., Thirion S., Jacques C. (2008). Primary culture and phenotyping of murine chondrocytes. Nat. Protoc..

[28] Jonason J.H., Hoak D., O’Keefe R.J., Westendorf J.J., van Wijnen A.J. (2015). Primary Murine Growth Plate and Articular Chondrocyte Isolation and Cell Culture. Osteoporosis and Osteoarthritis.

[29] Adkisson H.D., Martin J.A., Amendola R.L., Milliman C., Mauch K.A., Katwal A.B., Seyedin M., Amendola A., Streeter P.R., Buckwalter J.A. (2010). The potential of human allogeneic juvenile chondrocytes for restoration of articular cartilage. Am. J. Sports. Med..

[30] Alexopoulos L.G., Youn I., Bonaldo P., Guilak F. (2009). Developmental and osteoarthritic changes in Col6a1-knockout mice: Biomechanics of type VI collagen in the cartilage pericellular matrix. Arthritis Rheum..

[31] Di Cesare P.E., Fang C., Leslie M.P., Tulli H., Perris R., Carlson C.S. (2000). Expression of cartilage oligomeric matrix protein (COMP) by embryonic and adult osteoblasts. J. Orthop. Res..

[32] Sanchez C., Deberg M.A., Bellahcene A., Castronovo V., Msika P., Delcour J.P., Crielaard J.M., Henrotin Y.E. (2008). Phenotypic characterization of osteoblasts from the sclerotic zones of osteoarthritic subchondral bone. Arthritis Rheum..

[33] Frazer A., Bunning R.A., Thavarajah M., Seid J.M., Russell R.G. (1994). Studies on type II collagen and aggrecan production in human articular chondrocytes in vitro and effects of transforming growth factor-beta and interleukin-1beta. Osteoarthr. Cartil..

[34] Tsuda T., Markova D., Wang H., Evangelisti L., Pan T.C., Chu M.L. (2004). Zinc finger protein Zac1 is expressed in chondrogenic sites of the mouse. Dev. Dyn. Off. Publ. Am. Assoc. Anat..

[35] Rhee D.K., Marcelino J., Baker M., Gong Y., Smits P., Lefebvre V., Jay G.D., Stewart M., Wang H., Warman M.L. (2005). The secreted glycoprotein lubricin protects cartilage surfaces and inhibits synovial cell overgrowth. J. Clin. Investig..

[36] Kozhemyakina E., Zhang M., Ionescu A., Ayturk U.M., Ono N., Kobayashi A., Kronenberg H., Warman M.L., Lassar A.B. (2015). Identification of a Prg4-expressing articular cartilage progenitor cell population in mice. Arthritis Rheumatol..

[37] Bell D.M., Leung K.K., Wheatley S.C., Ng L.J., Zhou S., Ling K.W., Sham M.H., Koopman P., Tam P.P., Cheah K.S. (1997). SOX9 directly regulates the type-II collagen gene. Nat. Genet..

[38] Zhang P., Jimenez S.A., Stokes D.G. (2003). Regulation of human COL9A1 gene expression. Activation of the proximal promoter region by SOX9. J. Biol. Chem..

[39] Bridgewater L.C., Lefebvre V., de Crombrugghe B. (1998). Chondrocyte-specific enhancer elements in the Col11a2 gene resemble the Col2a1 tissue-specific enhancer. J. Biol. Chem..

[40] Sekiya I., Tsuji K., Koopman P., Watanabe H., Yamada Y., Shinomiya K., Nifuji A., Noda M. (2000). SOX9 enhances aggrecan gene promoter/enhancer activity and is up-regulated by retinoic acid in a cartilage-derived cell line, TC6. J. Biol. Chem..

[41] Akiyama H., Chaboissier M.C., Martin J.F., Schedl A., de Crombrugghe B. (2002). The transcription factor Sox9 has essential roles in successive steps of the chondrocyte differentiation pathway and is required for expression of Sox5 and Sox6. Genes Dev..

[42] Lui J.C., Yue S., Lee A., Kikani B., Temnycky A., Barnes K.M., Baron J. (2019). Persistent Sox9 expression in hypertrophic chondrocytes suppresses transdifferentiation into osteoblasts. Bone.

[43] Haseeb A., Kc R., Angelozzi M., de Charleroy C., Rux D., Tower R.J., Yao L., Pellegrino da Silva R., Pacifici M., Qin L. (2021). SOX9 keeps growth plates and articular cartilage healthy by inhibiting chondrocyte dedifferentiation/osteoblastic redifferentiation. Proc. Natl. Acad. Sci. USA.

[44] Lefebvre V., Li P., de Crombrugghe B. (1998). A new long form of Sox5 (L-Sox5), Sox6 and Sox9 are coexpressed in chondrogenesis and cooperatively activate the type II collagen gene. EMBO J..

[45] Han Y., Lefebvre V. (2008). L-Sox5 and Sox6 drive expression of the aggrecan gene in cartilage by securing binding of Sox9 to a far-upstream enhancer. Mol. Cell. Biol..

[46] Smits P., Li P., Mandel J., Zhang Z., Deng J.M., Behringer R.R., de Crombrugghe B., Lefebvre V. (2001). The transcription factors L-Sox5 and Sox6 are essential for cartilage formation. Dev. Cell.

[47] Outani H., Okada M., Yamashita A., Nakagawa K., Yoshikawa H., Tsumaki N. (2013). Direct induction of chondrogenic cells from human dermal fibroblast culture by defined factors. PLoS ONE.

[48] Stegen S., Rinaldi G., Loopmans S., Stockmans I., Moermans K., Thienpont B., Fendt S.M., Carmeliet P., Carmeliet G. (2020). Glutamine Metabolism Controls Chondrocyte Identity and Function. Dev. Cell.

[49] Iwamoto M., Yagami K., Lu Valle P., Olsen B.R., Petropoulos C.J., Ewert D.L., Pacifici M. (1993). Expression and role of c-myc in chondrocytes undergoing endochondral ossification. J. Biol. Chem..

[50] Varrault A., Dantec C., Le Digarcher A., Chotard L., Bilanges B., Parrinello H., Dubois E., Rialle S., Severac D., Bouschet T. (2017). Identification of Plagl1/Zac1 binding sites and target genes establishes its role in the regulation of extracellular matrix genes and the imprinted gene network. Nucleic Acids Res..

[51] Williams J.A., Kondo N., Okabe T., Takeshita N., Pilchak D.M., Koyama E., Ochiai T., Jensen D., Chu M.L., Kane M.A. (2009). Retinoic acid receptors are required for skeletal growth, matrix homeostasis and growth plate function in postnatal mouse. Dev. Biol..

[52] Valente T., Junyent F., Auladell C. (2005). Zac1 is expressed in progenitor/stem cells of the neuroectoderm and mesoderm during embryogenesis: Differential phenotype of the Zac1-expressing cells during development. Dev. Dyn. Off. Publ. Am. Assoc. Anat..

[53] Kamikihara T., Arima T., Kato K., Matsuda T., Kato H., Douchi T., Nagata Y., Nakao M., Wake N. (2005). Epigenetic silencing of the imprinted gene ZAC by DNA methylation is an early event in the progression of human ovarian cancer. Int. J. Cancer.

[54] Murakami S., Lefebvre V., de Crombrugghe B. (2000). Potent inhibition of the master chondrogenic factor Sox9 gene by interleukin-1 and tumor necrosis factor-alpha. J. Biol. Chem..

[55] Ouyang Y., Wang W., Tu B., Zhu Y., Fan C., Li Y. (2019). Overexpression of SOX9 alleviates the progression of human osteoarthritis in vitro and in vivo. Drug Des. Dev. Ther..

[56] Feng D., Kang X., Wang R., Chen H., Zhang K., Feng W., Li H., Zhu Y., Wu S. (2020). Progranulin modulates cartilage-specific gene expression via sirtuin 1-mediated deacetylation of the transcription factors SOX9 and P65. J. Biol. Chem..

[57] Lee J.S., Im G.I. (2011). SOX trio decrease in the articular cartilage with the advancement of osteoarthritis. Connect. Tissue Res..

[58] Xu J., Kang Y., Liao W.M., Yu L. (2012). MiR-194 regulates chondrogenic differentiation of human adipose-derived stem cells by targeting Sox5. PLoS ONE.

[59] Kuo C.L., Liu S.T., Chang Y.L., Wu C.C., Huang S.M. (2018). Zac1 regulates IL-11 expression in osteoarthritis. Oncotarget.

[60] Iqbal S.M., Leonard C., Regmi S.C., De Rantere D., Tailor P., Ren G., Ishida H., Hsu C., Abubacker S., Pang D.S. (2016). Lubricin/Proteoglycan 4 binds to and regulates the activity of Toll-Like Receptors In Vitro. Sci. Rep..

[61] Alquraini A., Jamal M., Zhang L., Schmidt T., Jay G.D., Elsaid K.A. (2017). The autocrine role of proteoglycan-4 (PRG4) in modulating osteoarthritic synoviocyte proliferation and expression of matrix degrading enzymes. Arthritis Res. Ther..

[62] Frisbie D.D., Morisset S., Ho C.P., Rodkey W.G., Steadman J.R., Mcllwraith C.W. (2006). Effects of calcified cartilage on healing of chondral defects treated with microfracture in horses. Am. J. Sport. Med..

[63] Yanke A.B., Lee A.S., Karas V., Abrams G., Riccio M.L., Verma N.N., Bach B.R., Cole B.J. (2019). Surgeon Ability to Appropriately Address the Calcified Cartilage Layer: An In Vitro Study of Arthroscopic and Open Techniques. Am. J. Sports. Med..

[64] Aghajanian P., Xing W., Cheng S., Mohan S. (2017). Epiphyseal bone formation occurs via thyroid hormone regulation of chondrocyte to osteoblast transdifferentiation. Sci. Rep..

