# Supplementary material for: Direct Reprogramming of Mouse Subchondral Bone Osteoblasts into Chondrocyte-like Cells
Source: Biomedicines. 2022 Oct 14;10(10):2582. doi: 10.3390/biomedicines10102582 (PMC9599480; doi:10.3390/biomedicines10102582)
Supplement: Supplementary file 1 [file biomedicines-10-02582-s001.zip › biomedicines-1871311-supplementary.pdf]

Supplementary Materials

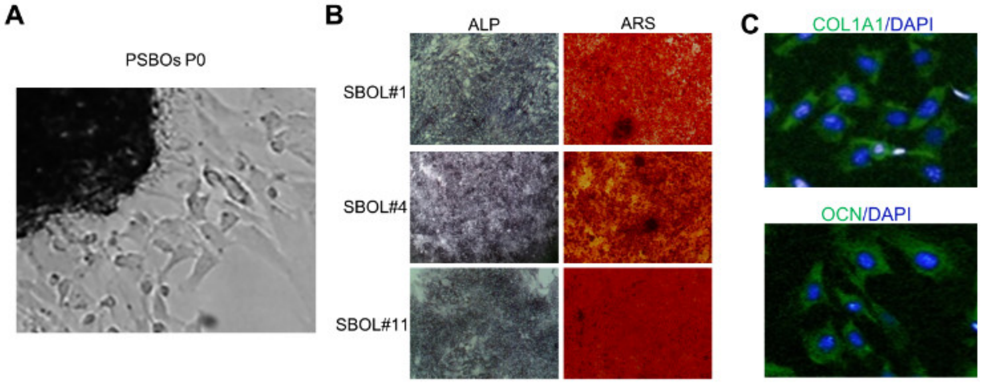

Figure S1. SBOLs retained osteoblastic properties. Subchondral bone osteoblasts (SBOLs) were selected in the presence of 10 μg/ml hygromycin B following immortalization of primary SBO (PSBO) cells by co-transfection with SV40 T Ag and piggyBac transposase expression vector. Subclones were selected by limiting dilution at one cell per well. (A) Light microscopy of PSBOs outgrowth from explants. Magnification: 100×. (B) Alkaline phosphatase (ALP) and Alizarin Red S (ARS) staining of SBOL subclones cultured for 7 and 12 d, respectively. Magnification: 40×. (C) Immunofluorescence microscopy of COL1A1 and OCN in SBOL clone #4. Magnification: 200×.

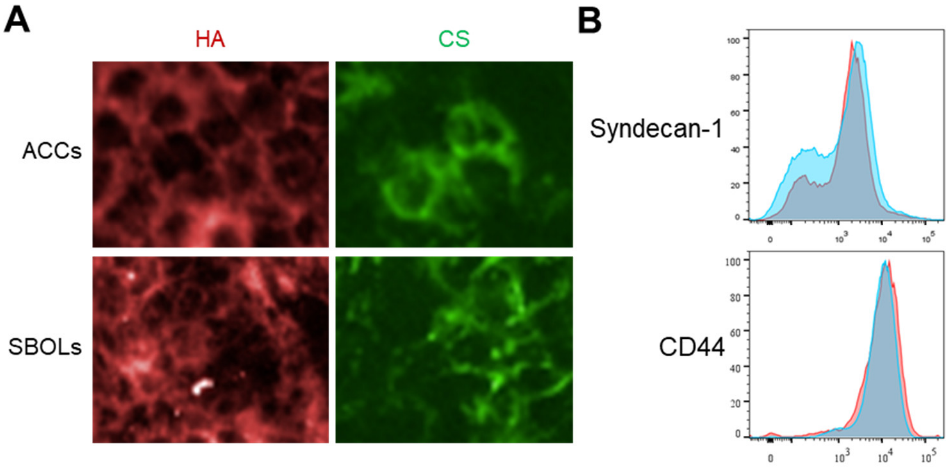

Figure S2. ACCs and SBOLs shared some indistinguishable features excluded for the characterization of cACCs. ACCs and SBOLs were cultured to confluence and then harvested for immunofluorescence and flow cytometric analyses. (A) Immunofluorescence microscopy of hyaluronan (HA) and chondroitin sulfate (CS). Magnification: 200×. (B) Flow cytometric analyses of syndecan-1 and CD44 on ACCs (red) and SBOLs (blue).

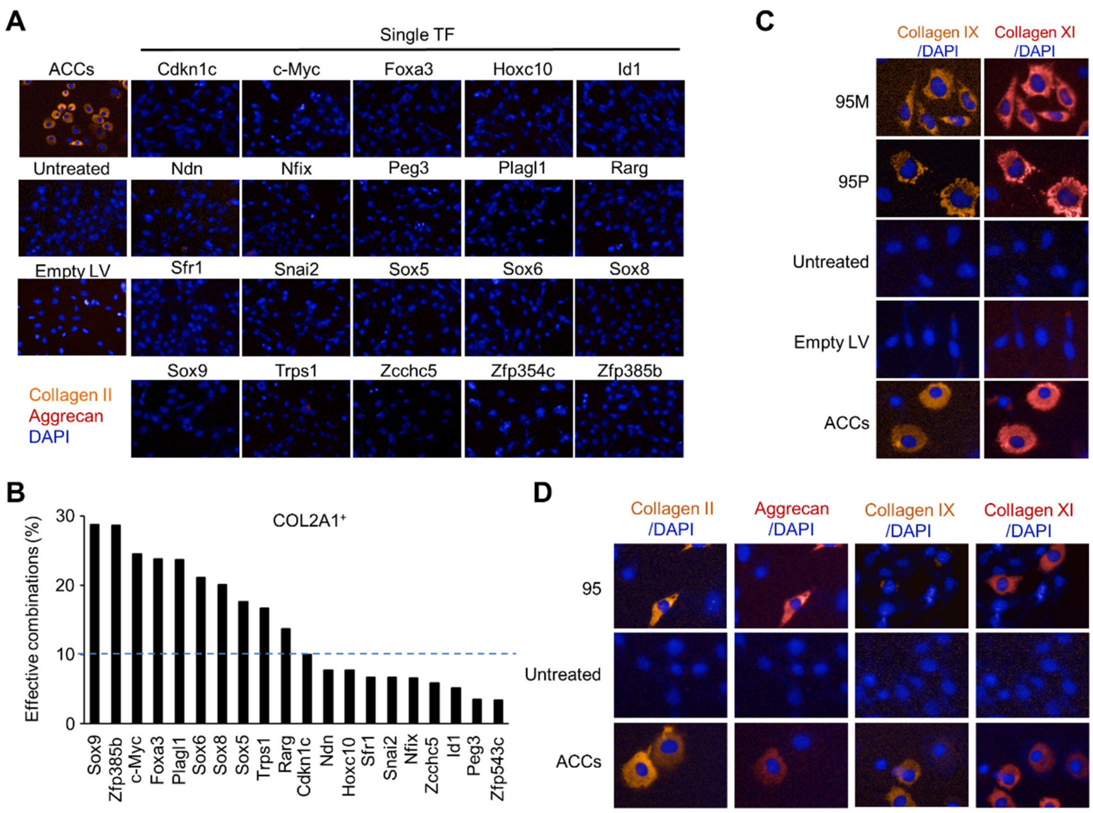

Figure S3. Sox9 and Sox5 together with c-Myc or Plagl1 as 3-TF combination was essential for cACCs generation. SBOLs were transduced with transcription-expressing lentiviruses at an MOI of 100 twice with 8 µg/ml polybrene. After 14 d of culture, cells were fixed for immunofluorescence analyses. (A) Immunofluorescence staining of COL2A1 and ACAN after transduction with single transcription factor indicated. Magnification: 100×. (B) Possibly effective combinations containing the indicated transcription factors, each of which was tested in at least 15 different combinations containing 6 transcription factors. The combination effectiveness was determined by COL2A1 expression. (C) Immunofluorescence staining of COL9A1 and COL11A1 in cells treated with either Sox9+Sox5+c-Myc or Sox9+Sox5+Plagl1. Magnification: 200×. (D) Immunofluorescence staining of collagens and aggrecan in Sox9+Sox5-treated cells. Magnification: 200×.

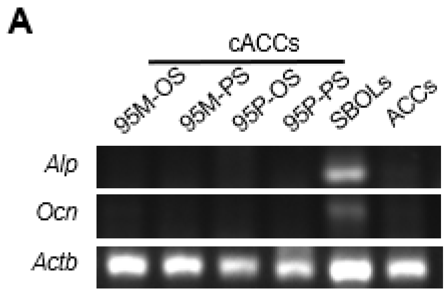

Figure S4. 95M- and 95P-reprogrammed SBOLs lost their osteoblast identity. SBOs were transfected with 95M or 95P and incubated for 14 d prior to the assessment of osteogenic related markers expression by PCR analysis in LCM cells based on their morphology (OS or PS) and chondrogenic phenotype (positive for both COL2A1 and ACAN examined under fluorescence microscope). The osteogenic markers *Alp* and *Ocn* were assessed. ACCs and SBOLs served as control.

Table S1. Sequences of primers used for lentivirus construction.

Gene	Accession		Sequence
Cdkn1c	NM_001354981.1	F:	TTAGCTAGCATGGGCATGTCCGACGTGTACCT
		R:	TTAGGATCCTCATCTCAGACGTTTGCGCGG
c-Myc	NM_010849.4	F:	TTAGCTAGCATGCCCCTCAACGTTAGCTTC
		R:	TTAGGATCCTTACGCACAAGAGTTCCGTAGCT
Foxa3	NM_008260.2	F:	TTAGCTAGCATGCTGGGCTCAGTGAAGATG
		R:	TTAGGATCCCTAGGATGCATTAAGCAGAGAGCG
Hoxc10	NM_010462.5	F:	TTAGCTAGCATGACATGCCCTCGCAATGTAAC
		R:	TTAACTAGTTCAGGTGAAATTAAAATTGGAGGTCA
Id1	NM_010495.3	F:	TTAGCTAGCATGAAGGTCGCCAGTGGCAGTGCC
		R:	TTAGGATCCTCAGTGCGCCGCCTCAGCGA
Ndn	NM_010882.3	F:	TTAGCTAGCATGTCGGAACAAAGTAAGGACCTGA
		R:	TTAGAATTCTTAGTCCTCAGAGACACTGCTGCG
Nfix	NM_001081982.3	F:	TTAGCTAGCATGTACTCCCCGTACTGCCTCAC
		R:	TTAACTAGTTCAGAAAGTTGCCGTCCCG
Peg3	NM_008817.2	F:	TTAGCTAGCATGTACCATCACGAAGACGACACCA
		R:	TTAGGATCCTCAACCAGTGTGAGAATTCTGGTGTCTG
Plagl1	NM_009538.3	F:	TTAGCTAGCATGGCTCCATTCCGCTGTCAA
		R:	TTAGGATCCTCAACCAGTGTGAGAATTCTGGTGTCTG
Rarg	NM_011244.4	F:	TTAGCTAGCATGGCCACCAATAAGGAGAGACTCTTTGC
		R:	TTAGGATCCTCAGGGCCCCTGGTCAGGTTGG
Sfr1	NM_026377.2	F:	TTAGCTAGCATGGCTGAGGAAGGAAATCAGGAG
		R:	TTAGGATCCTCACACCCCTGTAAACTCTTCTTCACTT
Snai2	NM_011415.3	F:	TTAGCTAGCATGCCGCGCTCCTTCCTGGT
		R:	TTAGGATCCTCAGTGTGCCACACAGCAGCCAG
Sox5	NM_011444.3	F:	GGCGCTAGCATGCTTACTGACCCTGATTTACCTC
		R:	GGCACTAGTTCAGTTGGCTTGTCCCGCA
Sox6	NM_011445.4	F:	GGCGCTAGCATGTCTTCCAAGCAAGCCACCT
		R:	GGCACTAGTACAAAAGCTCCTCAGTTGGCACT
Sox8	NM_011447.3	F:	TTAGAATTCATGCTGGACATGAGTGAGGCC
		R:	TTAGGATCCTCAGGGTCGGGTCAGGGTG
SOX9	Z46629.1	F:	TTAGCTAGCATGAATCTCCTGGACCCCTTCAT
		R:	TTAGGATCCTCAGGGTCTGGTGAGCTGTGTG
Trps1	NM_001310481.1	F:	GGCGCTAGCATGCAGAGTAATATGGTCCGGAAAAAGA
		R:	GGCACTAGTTTACTCTTTAGGTTTTCCATTTTTTTCTGC

Table S1. Cont.

Gene	Accession	Sequence
Zcchc5	NM_199468.2	F: TTAGCTAGCATGGTAGAGGACTTAGCAGCTTCCTATGT
		R: TTAACTAGTTTACCGCCGGGCCTCCATG
Zfp354c	NM_013922.4	F: TTAGCTAGCATGGCTGTGGATTTGCTGGCTG
		R: TTAACTAGTTCACAGCGATGAATTAAAGTTCTCAGG
Zfp385b	NM_001113400.1	F: TTAGCTAGCATGAACATGGCAACCTTCCTCC
		R: TTAGGATCCTTAGTAAGGGGCAAAGAGGATGGA

Table S2. Sequences of primers used in PCR reactions.

Gene	Accession		Sequence
Col2a1	NM_031163.3	F:	AGAGCGGAGACTACTGGATTGAT
		R:	TCATCTGGACGTTAGCGGTGTT
Col10a1	NM_009925.4	F:	CTTTGTGTGCCTTTCAATCG
		R:	GTGAGGTACAGCCTACCAGTTTT
Acan	NM_001361500.1	F:	GTTGGTTACTTCGCCTCCAG
		R:	GTCCTCCAAGCTCTGTGACC
Prg4	NM_021400.3	F:	TGGAGTGCTGTCCTGATTTCAAGAG
		R:	GGTGATTTGGGTGAGCGTTTGGTA
Col1a1	NM_007742.4	F:	AACCCGAGGTATGCTTGATCT
		R:	CCAGTTCTTCATTGCATTGC
Runx2	NM_001146038.2	F:	GACATCCCCATCCATCCACT
		R:	TGAGAGAGGAAGGCCAGAGG
Alp	NM_007431.3	F:	GTTGCCAAGCTGGGAAGAACAC
		R:	CCCACCCCGCTATTCCAAAC
Ocn	NM_007541.3	F:	GACCTCACAGATGCCAAGCC
		R:	AGGCGGTCTTCAAGCCATAC
Opn	NM_001204201.1	F:	CTGGCTGAATTCTGAGGGACT
		R:	TTCTGTGGCGCAAGGAGATT
Actb	NM_007393.5	F:	AGATGTGGATCAGCAAGCAG
		R:	GCGCAAGTTAGGTTTTGTCA

Table S3. Abbreviations.

Abbreviation	Full Name
AC	Articular cartilage
ACC	Articular cartilage chondrocyte
ALP	Alkaline phosphatase
ARS	Alizarin Red S
cACC	Transcription factor-converted ACC
CS	Chondroitin sulfate
DZ	Deeper zone
DZC	Deeper zone chondrocyte
ECM	Extracellular matrix
FBS	Fetal bovine serum
HA	Hyaluronan
LCM	Laser capture microdissection
LV	Lentivirus
OM	Osteogenic medium
OS	Oval shape
PCR	Polymerase Chain Reaction
Prg4	Proteoglycan 4
PS	Polygonal shape
PSBO	Primary subchondral bone osteoblast
RT-qPCR	Real-time–quantitative PCR
SB	Subchondral bone
SBO	Subchondral bone osteoblast
SBOL	Subchondral bone osteoblast cell line
SZ	Superficial zone
SZC	Superficial zone chondrocyte
TF	Transcription factor
95M-OS	Sox9+Sox5+c-Myc induced oval shaped cell
95M-PS	Sox9+Sox5+c-Myc induced polygonal shaped cell
95P-OS	Sox9+Sox5+Plagl1 induced oval shaped cell
95P-PS	Sox9+Sox5+ Plagl1 induced polygonal shaped cell
